# Redetermination of PD-L1 expression after chemio-radiation in locally advanced PDL1 negative NSCLC patients: retrospective multicentric analysis

**DOI:** 10.3389/fonc.2024.1325249

**Published:** 2024-01-31

**Authors:** Patrizia Ciammella, Salvatore Cozzi, Paolo Borghetti, Marco Galaverni, Valerio Nardone, Maria Paola Ruggieri, Matteo Sepulcri, Vieri Scotti, Alessio Bruni, Francesca Zanelli, Roberto Piro, Elena Tagliavini, Andrea Botti, Federico Iori, Emanuele Alì, Chiara Bennati, Marcello Tiseo

**Affiliations:** ^1^ Radiation Oncology Unit, Azienda USL-IRCCS di Reggio Emilia, Reggio Emilia, Italy; ^2^ Radiation Oncology Department, Centre Lèon Bèrard, Lyon, France; ^3^ Dipartimento di Radioterapia Oncologica, Università e ASST Spedali Civili di Brescia, Brescia, Italy; ^4^ Radiation Oncology Unit, University Hospital of Parma, Parma, Italy; ^5^ Dipartimento di Medicina di Precisione, Università degli Studi della Campania “L. Vanvitelli, Napoli, Italy; ^6^ Radiation Oncology Unit, Veneto Institute of Oncology IOV - IRCCS, Padua, Italy; ^7^ Oncology Department, Azienda Ospedaliero-Universitaria Careggi, Florence, Italy; ^8^ Radiation Therapy Unit, Department of Oncology and Hematology, University Hospital of Modena, Modena, Italy; ^9^ Oncology Unit, Azienda Unità Sanitaria Locale-IRCCS di Reggio Emilia, Reggio Emilia, Italy; ^10^ Pulmonology Unit, Azienda Unità Sanitaria Locale-IRCCS di Reggio Emilia, Reggio Emilia, Italy; ^11^ Pathology Unit, Azienda USL-IRCCS di Reggio Emilia, Reggio Emilia, Italy; ^12^ Medical Physics Unit, Azienda USL-IRCCS di Reggio Emilia, Reggio Emilia, Italy; ^13^ Clinical and Experimental Medicine PhD Program, Department of Biomedical, Metabolic, and Neural Sciences, University of Modena and Reggio Emilia, Modena, Italy; ^14^ Department of Hematology-Onco, S Maria delle Croci Hospital, Ravenna, Italy; ^15^ Department of Medicine and Surgery, University of Parma, Parma, Italy

**Keywords:** locally advanced non-small cell lung cancer, chemo-radiation, PD-L1 expression, PD-L1 negative patients, re-biopsy, durvalumab

## Abstract

**Background:**

Chemoradiation therapy (CRT) is the treatment of choice for locally advanced non-small cell lung cancer (LA-NSCLC). Several clinical trials that combine programmed cell death 1 (PD1) axis inhibitors with radiotherapy are in development for patients with LA-NSCLC. However, the effect of CRT on tumor cells programmed cell death ligand-1 (PD-L1) expression is unknown.

**Methods:**

In this multicentric retrospective study, we analyzed paired NSCLC specimens that had been obtained pre- and post-CRT. PD-L1 expression on tumor cells was studied by immunohistochemistry. The purpose of this study was to evaluate the feasibility, risk of complications, and clinical relevance of performing re-biopsy after CRT in patients with PD-L1 negative LA-NSCLC.

**Results:**

Overall, 31 patients from 6 centers with PD-L1 negative LA-NSCLC were analyzed. The percentage of tumor cells with PD-L1 expression significantly increased between pre- and post-CRT specimens in 14 patients (45%). Nine patients had unchanged PD-L1 expression after CRT, in five patients the rebiopsy material was insufficient for PD-L1 analysis and in two patients no tumor cells at rebiopsy were found. The post-rebiopsy complication rate was very low (6%). All patients with positive PD-L1 re-biopsy received Durvalumab maintenance after CRT, except one patient who had a long hospitalization for tuberculosis reactivation. Median PFS of patients with unchanged or increased PD-L1 expression was 10 and 16.9 months, respectively.

**Conclusion:**

CRT administration can induce PD-L1 expression in a considerable fraction of PD-L1 negative patients at baseline, allowing them receiving the maintenance Durvalumab in Europe. Hence, after a definitive CRT, PD-L1 redetermination should be considered in patients with LA-NSCLC PD-L1 negative, to have a better selection of maintenance Durvalumab candidates.

## Introduction

Non-small cell lung cancer (NSCLC) is the second most common cancer and a leading cause of cancer-related death worldwide, with approximately 25% of patients diagnosed with a locally advanced disease (1). Historically the standard of care for patients with a good performance status and unresectable locally advanced NSCLC (LA-NSCLC) has been a platinum-based doublet chemotherapy in combination with a radiation treatment (namely, chemoradiotherapy: CRT). However, the median progression-free survival among patients who have received CRT was poor (approximately 8 months), and only 15% of patients were alive at 5 years (2–4). Although the attempts to improve patient’s survival by associating different new drugs with CRT, the results were disappointing until the results of Pacific trial (5–11). In fact, the randomized phase 3 PACIFIC trial established a new standard for unresectable LA-NSCLC, introducing the concept of immunotherapy maintenance with the anti-programmed-death ligand 1 (anti-PD-L1) agent Durvalumab, for patients without progressive disease after CRT. The maintenance with Durvalumab, administered for up to 12 months after CRT, increase both Overall Survival (OS: 43.5 *vs* 29.1 months at 3 years) and Progression-Free Survival (PFS) compared to placebo, with a low immune-related side events of any grade (25%) (10, 11).

PD-L1 is expressed by cells in the tumor microenvironment, and it engages PD-1 on T cells. It triggers inhibitory signaling of the T cell receptor, reducing T-cell killing capacity and blocking effector functions (12). However, the PD-L1 expression on tumor cells is dynamic and it can be induced by the administration of oncological treatments, such as CRT. There is evidence that in patients with NSCLC, who underwent neoadjuvant CRT followed by surgery, a significant increase in PD-L1 expression was determined (13). Considering this, re-biopsy after CRT may better select PD-L1 positive patients. Arguments against re-biopsy include the risk of complications with the likelihood of getting an insufficient amount of tumor tissue for analyses. Notwithstanding this, performing PD-L1 re-determination after CRT in patient resulted PDL1 negative at the beginning, can allow offering maintenance Durvalumab to patients who otherwise could have not benefit from it, as the European Medicines Agency (EMA) has recommended Durvalumab exclusively in patients with a tumor proportion score PDL1 (TPS)‗ 1%. Thus, although the Food and Drug Administration (FDA) and other pharmaceutical agencies have approved the use of Durvalumab in all patients, regardless of PD-L1 expression, PD-L1 negative patients cannot receive it in Europe. The EMA based this on a *post-hoc* analysis, which showed that patients with tumors that did not express PD-L1 had no survival advantage over control.

Thus, the aim of this retrospective multicentric study was to analyze paired NSCLC specimens pre- and post-CRT, in patients with inoperable LA-NCSLC, PD-L1 negative at diagnosis, to explore the impact of CRT on PD-L1. Additionally, this work aims to evaluate the feasibility, the risk of complications, and the clinical relevance for performing re-biopsy systematically after CRT, in patients with PD-L1 negative LA-NSCLC.

## Methods

We have retrospectively evaluated patients with PD-L1 negative unresectable LA-NCSLC who undergoing CRT and subsequently re-biopsy for the re-determination of PD-L1 in 10 Italian centers.

Inclusion criteria were: 1) patients over 18 years of age; 2) histological diagnosis of unresectable LA-NSCLC; 3) negative PD-L1 expression tested before the start of CRT; 4) concurrent or sequential CRT; 5) no progressive disease at early evaluation after CRT; 6) re-biopsy of the tumor (primary tumor or mediastinal lymphadenopathies); 6) signature of informed consent. Exclusion criteria were following: 1) patients with stage IV NSCLC; 2) patients with Small Cell Lung Cancer; 3) absence of pre-treatment PD-L1 expression determination; 4) progressive disease after CRT; 5) previous thoracic irradiation; 6) diagnosis of other concurrent cancer except for non-melanomatous skin cancers; A diagnostic biopsy was performed at baseline (at diagnosis) and repeated after CRT.

All patient’s clinical characteristics (age, sex, smocking habitus), disease data (histological type, TNM stage, genomic aberrations, PD-L1 expression before and after CRT), treatment details (type of concurrent drugs, radiotherapy doses and fractionation) and clinical outcomes data (overall survival, progression free survival) were collected in anonymous database. Additionally, the re-biopsy modalities and complications were recorded. Complications were defined as severe if required hospitalization.

The primary endpoint was to evaluate the variation in PD-L1 expression before and after CRT in patients with LA-NSCLC. Secondary objectives were clinical relevance, defined as a potential of changing treatment, due to new histological evidence, specifically a change in PD-L1 TPS from negative (<1%) to positive (>1%) (possibility of administering maintenance Durvalumab), acute complication rate to re-biopsy, rate of non-diagnostic procedure (including negative ones or insufficient material to test PDL1), progression free survival (PFS) and overall survival (OS).

PD-L1 expression was examined by staining on the Dako Autostainer 48 (Dako Omnis platform) using the PD-L1 IHC 22C3 pharmDx kit. The percentage of PD-L1 positive tumor cells were evaluated by expert pathologists, blinded to clinical outcome. TPS was calculated as the percentage of tumor cells showing partial or complete membrane staining relative to all viable tumor cells in the sample. Based on staining intensity, a division into four main groups was performed: negative (<1%), weak (≥1% or <5%), moderate (≥5% or <50%) and strong (≥50%). No PD-L1 TPS was performed if no malignant cells or only suspected malignant cells were found. The present study received final approval by the Institutional Ethical Committee and was performed in accordance with the principles of Good Clinical Practice (GCP) in respect of the ICH GCP guidelines and the ethical principles contained in the Helsinki declaration and its subsequent updates. A written consent form was obtained from each patient.

For clinical and pathological characteristics, descriptive statistics were applied and presented as frequencies, percentages, and median (range). Time to re-biopsy was calculated as the interval from end of CRT to the performance of biopsy (days). PFS was defined as the time from CRT initiation to radiologically verified progression. Patients with no progression by the cut-off date of August 30, 2022 were listed. Overall survival was calculated from date of first-line treatment initiation to date of death or until a cut-off date of August 30th, 2022. The survival curves were calculated by the Kaplan-Meier method and differences in survival were tested by the log-rank test. Univariate analyses were performed with a log rank test. Statistics were performed using SPSS 20.0 software (Chicago, IL) and the significance level was set at P-value (2-sided) <0.05.

## Results

From January 2019 to January 2022, 31 consecutive patients, from 6 Italian centers, met the inclusion criteria and were enrolled in present study. All enrolled patients were PD-L1-negative LA-NSCLC patients at diagnosis and all were treated with up-front CRT (concurrent or sequential). With the exception of one patient, whose rebiopsy was postponed due to an acute cardiac event, all other 30 patients (97%) were investigated after up-front CRT.

Patient characteristics are summarized in **Table 1**. Among them, most patients were male (81%) and smokers (90%); 13 patients had stage IIIA disease, while 12 patients had stage IIIB and 6 pts IIIC disease, respectively. Nine patients had squamous histology, while 22 had non squamous NSCLC. Most patients (48%) received taxolo plus carboplatin as the CRT regimen.

**Table 1:** Characteristics of the 31 patients with both pre- and post-CRT specimens.

Patient characteristic	Total (%) (N = 31)
Age (years)	
Range	49-78
Mean	64.3
Sex	
Male	81% (25)
Female	19% (6)
Smoking status	
never-smoker	9.6% (3)
current or former smoker	90.4% (28)
Histology	
Non squamous	71% (22)
Squamous	29% (9)
Stage	
III A	42% (13)
III B	39% (12)
III C	19% (6)
ECOG-PS	
0	81% (25)
1	19% (6)
CRT regimen	
Taxolo plus platinum	48% (15)
Vinorelbine plus platinum	6% (2)
Pemetrexed plus platinum	6% (2)
Etoposide plus platinum	32% (10)
Gemcitabine plus platinum	6% (2)
Radiotherapy dose	
< 50 Gy	0
50-59 Gy	13% (4)
= 60 Gy	87% (27)
> 60 Gy	0
CRT timing	
Sequential	23% (7)
Concurrent	77% (24)
Radiological response after CRT	
Complete response	0
Partial Response >50%	35% (11)
Partial response ≤ 50%	42% (13)
Stable disease	23% (7)
Progression disease	0
PD-L1 status on tumor cells before CRT	
Negative	100% (31)
Biopsy procedures	
Broncoscopy +/- EBUS/EUS	78% (24)
Transtoracic biopsy	22% (7)
Biopsy site	
Primary Tumor	52% (16)
Mediastinal lymphoadenopaties	26% (8)
Both	22% (7)

ECOG PS, Eastern Cooperative Oncology Group Performance Status; PDL1, programmed cell death ligand-1; CRT, chemo-radiation therapy.

Re-biopsy characteristics and results are reported in **Table 2**. The median time between the last day of RT and re-biopsy was 31 days (15–56). Among the 24 patients with sufficient material after CRT, 14 (58%) had positive PD-L1 expression on tumor cells in the post-CRT specimens. The positivization rate was 45% (95%CI: 35-55). In 9 patients PD-L1 expression was negative in both the pre- and post-CRT specimens. In five patients the rebiopsy material was insufficient for PD-L1 analysis and in two patients no tumor cells at rebiopsy were found.

**Table 2:** Re-biopsy characteristics and results.

Re-biopsy procedures	
Broncoscopy +/- EBUS/EUS	84% (26)
Transtoracic biopsy	16% (5)
Re-biopsy site	
Primary tumor	48% (15)
Mediastinal lymphoadenopaties	39% (12)
Both	13% (4)
Time from RT-end to re-biopsy (days)	
Range	15-56
Median	31
PD-L1 status on tumor cells after CRT	
Positive	45% (14)
Negative	32% (10)
Undetermined^*^	23% (7)

^*^ 5 patients with insufficient rebiopsy material for PD-L1 analysis and two patients with no tumor cells at rebiopsy. PDL1, programmed cell death ligand-1; CRT, chemo-radiation therapy.

The location of re-biopsy was primary lung tumor, mediastinal lymphadenopathies or both in 15 (48%), 12 (39%) and 4 (13%) patients, respectively. The location of the re-biopsy corresponded to the site of the original biopsy (same anatomic sites) in 28 patients. In 3 patients (9.6%), the re-biopsy was taken from another location than the diagnostic biopsy. In 26 patients, re-biopsies were performed at the “pulmonary endoscopy unit” with bronchoscopy (lung lesions), endoscopic ultrasound of peri-bronchial/-tracheal (endobronchial ultrasound, EBUS) or para-gastroesophageal (endoscopic ultrasound, EUS - either with the EBUS endoscope (EUS-B) or conventional EUS-scope) structures. Transthoracic sampling from lung lesions is performed in 5 patients. Re-biopsy modalities are reported in **Table 2**.

No severe acute complications occurred during the re-biopsy in any of the patients. The rate of overall complications to rebiopsy after CRT was 6% (n = 2, 1 pneumothorax and 1 bronchial hemorrhage). No severe late complications or subsequent sequelae occurred in any of the patient cases.

A potential clinical relevance of re-biopsy was obtained in 14 out of the re-biopsied 31 patients (45%); in fact, in the 14 patients in which a change from PD-L1 TPS negative to positive was highlighted, it was possible to administer Durvalumab maintenance (one patient was unable to take Durvalumab due to concurrent reactivated tuberculosis).

**Figure 1:**
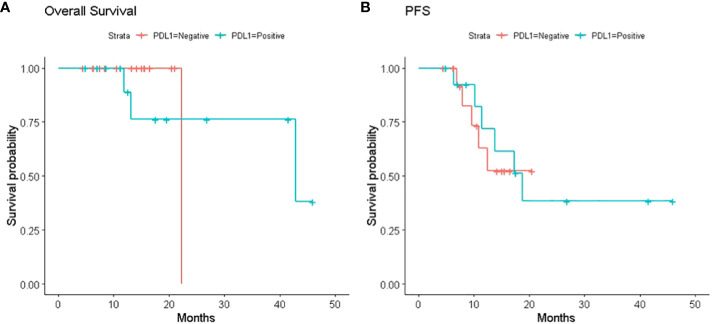
**(A)** Kaplan–Meier survival curves. Kaplan–Meier survival curves of overall survival (OS) in patients with or without PDL1 expression on tumor cells in the post-CRT specimens; **(B)** Kaplan–Meier survival curves of progression free survival (PFS) in patients with or without PDL1 expression on tumor cells in the post-CCRT specimens.

We also investigated the association between the change in PD-L1 expression and survival time. These survival curves are shown in **Figure 1**. Among the 24 patients with sufficient post-CRT material, PD-L1 expression in post-CRT material was associated with moderate increase of PFS (PD-L1-positive group versus PD-L1-negative group, median 16.9 versus 10 months respectively, p = 0.6) without difference in median OS (PD-L1-positive group versus PD-L1-negative group, median 20 versus 14 months, p >0.5, respectively). 13 patients out of 14 with positive PD-L1 post-CRT (93%) received Durvalumab maintenance during the observation period. In these patients the median PFS and OS were 17.7 and 21.3 months, respectively. No patient was positive for EGF-R while the ALK rearrangement and the KRAS mutation were present in one patient each, only.

## Discussion

In the PACIFIC trial of unresectable LA-NSCLC patients whose disease had responded or stabilized after CRT, Durvalumab significantly improved PFS and OS (10). These results have led to the growing recognition of the ‘PACIFIC regimen’ (Durvalumab after CRT) as the standard of care in this setting, and to global approvals of Durvalumab for treatment of patients with unresectable, LA-NSCLC in the absence of disease progression following platinum-based CRT (14–16). However, in Europe, based on the results of *post hoc* analyses requested by the European Medicines Agency (EMA), patients must also have tumors that express PD-L1 on >1% of tumor cells (TCs) (17).

The field of research in PD-L1 TPS changes in NSCLC is mainly dominated by retrospective studies including patients with localized/resectable disease, having received neo-adjuvant or adjuvant chemotherapy (18–24), but the results remain conflicting and controversial. For example, Sheng et al. reported that the positivity of PD-L1 from 75% to 37.5% after neoadjuvant chemotherapy in NSCLC (18), while Rojkó revealed that PD-L1 expression showed no significant changes after neoadjuvant chemotherapy in patients with lung cancer (25). Song et al. demonstrated that the expression of PD-L1 could be upregulated by neoadjuvant chemotherapy in lung squamous cell carcinoma patients (19) as well as in Guo et al. ‘s study (26).

To our knowledge, however, there are no prospective studies that have investigated the role of rebiopsy after CRT in LA-NSCLC, either in terms of feasibility or clinical relevance. A retrospective study on 35 patients with LA-NSCLC, with paired NSCLC specimens that had been obtained pre- and post-CRT, showed that the percentage of PD-L1-positive tumor cells significantly decreased after CRT (27).

Our multicentric retrospective studies showed that PD-L1 TPS expression can be induced by CRT administration in approximately half of the PD-L1 TPS patients resulted negative at the baseline. Consequently, the execution of a re-biopsy in this group of patients may increase the number of candidates to maintenance Durvalumab according to EMA criteria, due to change in PD-L1 TPS expression from negative (<1%) to positive (>1%). Our data suggest that a re-biopsy is feasible, with the use of various biopsy-modalities, safe, as no severe complications were recorded, and with a high success-rate. This latter data is interesting as biopsies containing insufficient tumor tissue with none or too few tumor cells to perform molecular analysis was previously reported as a challenge by Chouaid and colleagues in 18% and 7% of cases, respectively (28).

Differences in PD-L1 TPS between tumor sites have been explored in several studies and a general consensus of both inter- and intra-tumoral heterogeneity have been established, above all in stage IV NSCLC (29–32). Almost all of these studies evaluated the concordance rate of PD-L1 expression between primary and metastatic tumor sites in stage IV NSCLC and report a high concordance for tumors with a PD-L1 TPS of <1% or ≥50% (32). A recent work confirms these data, and the authors point out how, due to the known and widely explored heterogeneity of PD-L1 expression, it could be questioned if the changes in PD-L1 TPS observed, could solely be explained by a different location of re-biopsy. These authors found a change in PD-L1 TPS with nearly the same incidence in patients who had a re-biopsy performed at the same or another location as the diagnostic biopsy (33). Our numbers are too small to determine whether a given biopsy procedure rather than the biopsied site is at greater risk of insufficient material; but it certainly makes sense that more biopsies can increase the success rate of the procedure.

This data is interesting considering the fear of performing biopsies in previously irradiated areas and that up to 40% of patients do not have tumor biopsies suitable for histological PD-L1 assessment at baseline (e.g., due to inadequate tissue collection using fine needle aspiration), and cytological assessment of PD-L1 expression, while feasible, is not yet widely standardized in routine clinical practice (34). Without considering patients who have negative PD-L1 and are denied access to maintenance Durvalumab after CRT.

The current study has some limitations. First, it is a retrospective study with a relatively small sample. Second, considering the great variability and the several problems in assessing PD-L1 expression, it is important to underline that no centralized review of either primary biopsies or re-biopsies was performed. Third, we did not investigate PD-L1 expression with the fluorescent *in situ* hybridization (FISH), generally considered more reliable.

## Conclusion

Re-biopsy is often offered to patients with progressive stage IV NSCLC after the first-line therapy, as it can provide important biological information to guide the second-line treatment decisions. However, despite its potential clinical advantages, performing a re-biopsy is not mandatory or regularly incorporated into the daily clinical practice.

Our study showed that re-biopsy is feasible, with low risk of complications, and can be clinically relevant in patients with LA- NSCLC PD-L1 negative. Thus, PD-L1 redetermination should be considered after a definitive CRT, in patients with LA-NSCLC PD-L1 negative, as it may allow them receiving the maintenance Durvalumab. Certainly, future prospective studies are needed to validate our results.

## Data availability statement

The datasets presented in this study can be found in online repositories. Access to the raw data and names of the repositories should be directed to Patrizia Ciammella (patrizia.ciammellal@ausl.re.it).

## Ethics statement

Ethical approval was not required for the studies involving humans because this is a clinical practice activity with no management of single data and possible recognition of data and patient. The studies were conducted in accordance with the local legislation and institutional requirements. The human samples used in this study were acquired from a by- product of routine care or industry. Written informed consent to participate in this study was not required from the participants or the participants’ legal guardians/next of kin in accordance with the national legislation and the institutional requirements.

## Author contributions

PC: Conceptualization, Data curation, Investigation, Writing – original draft, Writing – review & editing. SC: Data curation, Software, Writing – review & editing. PB: Writing – review & editing. MG: Writing – review & editing. VN: Writing – review & editing. MR: Data curation, Writing – review & editing. MS: Writing – review & editing. VS: Writing – review & editing. ABr: Data curation, Formal analysis, Software, Writing – review & editing. FZ: Writing – review & editing. RP: Writing – review & editing. ET: Writing – review & editing. ABo: Data curation, Formal analysis, Software, Writing – review & editing. FI: Data curation, Writing – review & editing. EA: Data curation, Writing – review & editing. CB: Writing – review & editing. MT: Writing – review & editing.
